# Characterization of siRNAs clusters in *Arabidopsis thaliana* galls induced by the root-knot nematode *Meloidogyne incognita*

**DOI:** 10.1186/s12864-018-5296-3

**Published:** 2018-12-18

**Authors:** Clémence Medina, Martine da Rocha, Marc Magliano, Alizée Raptopoulo, Nathalie Marteu, Kevin Lebrigand, Pierre Abad, Bruno Favery, Stéphanie Jaubert-Possamai

**Affiliations:** 1 INRA, Université Côte d’Azur, CNRS, ISA, Paris, France; 2 UCA Genomix, Institut de Pharmacologie Moléculaire et Cellulaire, CNRS UMR6097, Sophia Antipolis, Nice, France

**Keywords:** Gall, Giant cell, siRNA, Plant parasitic nematodes, Small RNA, Transcriptome regulation, Transposable element

## Abstract

**Background:**

Root-knot nematodes (RKN), genus *Meloidogyne*, are plant parasitic worms that have the ability to transform root vascular cylinder cells into hypertrophied, multinucleate and metabolically over-active feeding cells. Redifferentiation into feeding cells is the result of a massive transcriptional reprogramming of root cells targeted by RKN. Since RKN are able to induce similar feeding cells in roots of thousands of plant species, these worms are thought to manipulate essential and conserved plant molecular pathways.

**Results:**

Small non-coding RNAs of uninfected roots and infected root galls induced by *M. incognita* from *Arabidopsis thaliana* were sequenced by high throughput sequencing. SiRNA populations were analysed by using the Shortstack algorithm. We identified siRNA clusters that are differentially expressed in infected roots and evidenced an over-representation of the 23–24 nt siRNAs in infected tissue. This size corresponds to heterochromatic siRNAs (hc-siRNAs) which are known to regulate expression of transposons and genes at the transcriptional level, mainly by inducing DNA methylation.

**Conclusions:**

Correlation of siRNA clusters expression profile with transcriptomic data identified several protein coding genes that are candidates to be regulated by siRNAs at the transcriptional level by RNA directed DNA methylation (RdDM) pathway either directly or indirectly via silencing of neighbouring transposable elements.

**Electronic supplementary material:**

The online version of this article (10.1186/s12864-018-5296-3) contains supplementary material, which is available to authorized users.

## Background

Small RNAs (sRNAs) are 20- to 24-nucleotide (nt) non-coding RNAs that regulate gene expression at the transcriptional and post-transcriptional levels in eukaryotes [1]. There are two principal classes of small RNAs in plants, classified according to their biogenesis: microRNAs (miRNAs) and small interfering RNAs (siRNAs) [2, 3]. MicroRNAs are 21–22 nt long and are produced from a single-strand RNA precursor folded into a hairpin. Plant miRNAs are well characterised. They induce post-transcriptional gene silencing (PTGS) principally by triggering messenger RNA (mRNA) degradation, but they can also induce translational repression [4]. By contrast, siRNAs are 21–24 nt long and are produced from double stranded RNA (dsRNA) precursors resulting from (i) the hybridisation of two complementary RNA strands or (ii) de novo synthesis from a single-stranded RNA as a new complementary strand by RNA-dependent RNA polymerases (RDRs) [5, 6]. siRNAs mediate gene repression at the transcriptional or post-transcriptional level and form several classes differing in terms of predominant size [3].

Transcriptional gene silencing (TGS) takes place in two phases with different specific actors. The pre-establishment phase involves a DNA-dependent RNA polymerase (Pol) II producing aberrant transcripts, RDR6, which produces dsRNAs that are processed by Dicer-like protein 2 (DCL2) and DCL4 to produce 21–22 nt siRNAs that induce DNA methylation by DOMAINS REARRANGED METHYLTRANSFERASE (DRM) 2 and, probably, DRM1, through Argonaute 6 - RNA Induced Silencing Complex (AGO6-RISC) and PolV transcripts [7, 8]. Once RdDM has been established, a stabilisation phase occurs. This phase involves single-strand RNA transcripts produced by PolIV from intergenic or repetitive regions of the genome, which are rendered double-stranded by RDR2 and processed by DCL3 to produce 24 nt hc-siRNAs. Hc-siRNAs are loaded onto AGO4-RISC to initiate RdDM through the DRM2 and, probably, DRM1 proteins at hc-siRNA-generating loci, using other transcripts produced by the DNA-dependent Pol V [8]. One of the main functions of RdDM is maintaining genome integrity, by ensuring that suppressive levels and types of DNA methylation are maintained at transposable elements. RdDM may also modulate the expression of neighbouring protein-coding genes through the spread of DNA methylation [9–12].

siRNAs are also involved in PTGS. They are loaded onto AGO1 or AGO2-RISC, where they generally either induce the cleavage of target transcripts or prevent their translation [3]. The siRNAs involved in PTGS can be classified into several subfamilies according to the origin of the precursor. Natural antisense siRNAs (nat-siRNAs) are generated by the processing of dsRNA precursors derived from endogenous RNAs with complementary sequences, through the action of DCL4 or DCL2, to generate sRNAs of 21 and 22 nt in length, respectively. Phased, secondary, small interfering RNAs (phasiRNAs) are mostly 21 nt siRNAs derived from a RNA converted to dsRNA by RDR6 and processed by DCL4. One well characterised family of phasiRNAs are *Arabidopsis trans*-acting siRNAs (tasiRNAs) [13, 14]. The production of double-stranded phasiRNA precursors is stimulated by one or more upstream miRNAs, such as *TAS3*-derived tasiRNAs in *A. thaliana,* which are generated from non-coding *TAS3* transcripts by miR390 triggers [15, 16].

In plants, siRNAs have been shown to regulate gene expression in various biological processes, including growth, development [15], cell differentiation [17], and plant responses to abiotic and biotic stresses [18–21]. Various PTGS-inducing siRNAs (21–22 nt) have been shown to be related to plant immunity. Examples include nat-siRNAATGB2 from *A. thaliana,* which is induced by *Pseudomonas syringae* pv*. tomato* and plays a positive role in disease resistance by repressing the pentatricopeptide repeats protein–like gene [22]. The nucleotide-binding leucine-rich repeat (NB-LRR) gene family is widely targeted by secondary siRNAs, and phasiRNAs derived from NBS-LRRs play a key role in regulating plant immunity [14, 23]. For example, in Arabidopsis, miR472 and its RDR6-mediated gene silencing help to modulate both PAMP-triggered (PTI) and effector-triggered (ETI) immunity [24]. TGS-mediating hc-siRNAs have also emerged as major regulators of plant immunity directing DNA methylation and/or histone modification. A role for hc-siRNA-mediated TGS in plant immunity has also come to light, as fungal elicitors induce alterations in the accumulation of certain hc-siRNAs [12]. Moreover, the RdDM machinery has been shown to be involved in plant responses to several pathogens, including *Botrytis cinerea, P. syringae,* and *Agrobacterium tumefaciens* [25–27].

Root-knot nematodes (RKN), *Meloidogyne* spp., are highly polyphagous sedentary plant parasites capable of infesting most crop species [28, 29]. After penetrating host roots, RKN larvae migrate toward the vascular cylinder and reprogram gene expression in several vascular root cells, to induce their development into hypertrophied multinucleate giant feeding cells (GCs) [30]. These GCs are metabolically overactive, and serve as the sole source of the nutrients required for RKN development. The growth of the GCs and divisions of the surrounding cells lead to a root deformation known as a knot or gall. The redifferentiation of vascular cells into GCs results from the extensive reprogramming of gene expression in root cells, in response to RKN signals [31]. The expression of genes encoding proteins involved in metabolism, the cytoskeleton, cell cycle, cell rescue, defence, hormones, cellular communication and cellular transport are modified in galls and giant cells from various plant species [30, 32–35]. We are beginning to decipher the genetic pathways modified in infected roots for the formation of GCs, but little is known about the factors regulating this reprogramming of gene expression.

To date, the role of siRNAs in plant-nematode interactions has been little investigated. Two studies provided a first general overview of the small RNA populations expressed in the early feeding sites induced in *A. thaliana* roots by the RKN *M. javanica* [36] and the beet cyst nematode (CN) *Heterodera schachtii* [37]. The first detailed analysis of plant siRNAs expressed in response to *H. schachtii* identified 125 putative *A. thaliana* siRNAs expressed in root feeding sites named syncytia. The methylome of *A. thaliana* syncytia and the associated population of 24 nt siRNAs were recently studied and their abundance was found to be associated with the hypermethylation of transposable elements (TEs) and gene promoters [38]. Moreover, an analysis of the length distribution of Arabidopsis sRNAs in the early developing galls induced by *M. javanica* showed markedly larger numbers of 24 nt reads than in uninfected roots and an overexpression of *miR390* and its secondary *TAS3*-siRNA in galls [36]. Resistance to RKN of Arabidopsis mutant lines for *TAS3a* confirmed the role of these small RNAs in the plant-RKN interaction via the control of Auxin Responsive Factor 3 expression. Overall, these analyses suggest that plant parasitic nematodes use both microRNAs and the siRNA pathway to manipulate host gene expression at the transcriptional and/or post-transcriptional levels.

We developed a sequencing strategy to characterise the siRNA populations expressed in galls at two key points in giant cell/gall development: 7 days post infection (dpi), corresponding to the phase of successive nuclear divisions without cytokinesis; and 14 dpi, corresponding to the phase of isotropic growth and an increase in DNA levels through endoreduplication without nuclear divisions [39]. In this study, we focused on genomic regions named clusters corresponding to accumulations of siRNAs differentially expressed (DE) between galls and uninfected roots. This analysis provides insight into the loci targeted by siRNAs during the plant-nematode interaction (coding genes, promoting regions, transposable elements). We then identified differentially expressed genes corresponding to differentially expressed clusters with inversely correlated expression profiles, providing biological support for the regulation of these genes by siRNA pathways.

## Results

### Identification of predicted siRNA clusters in galls and uninfected roots

**Fig. 1:**
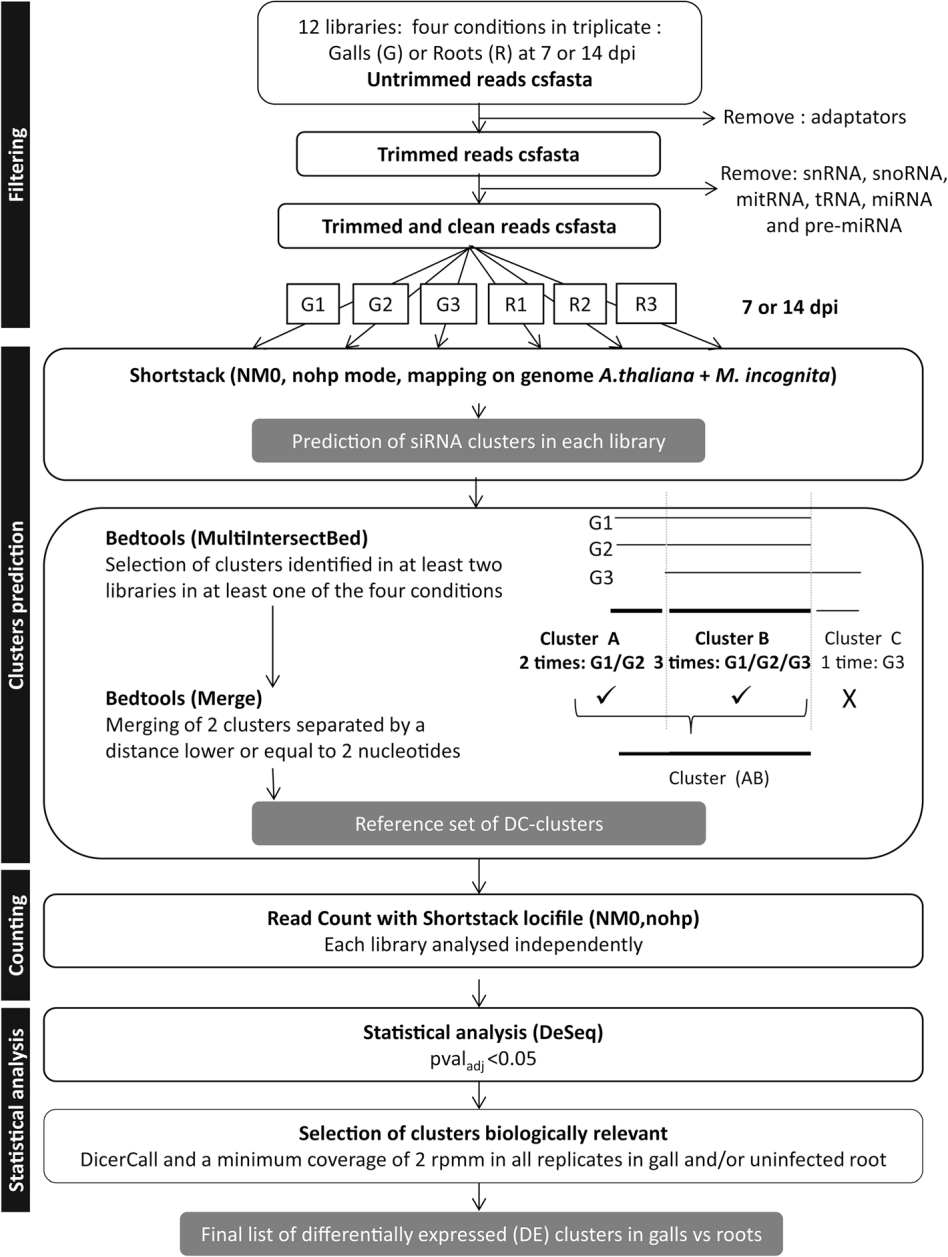
Pipeline of siRNA analysis from prediction to statistical analysis. Data obtained from the SOLiD sequencing of the 12 libraries were cleaned of adaptors and special sequences (snRNA, snoRNA, mitRNA, tRNA, miRNA and pre-miRNA). The Shortstack algorithm mapped and identified clusters corresponding to genomic regions accumulating siRNAs. The algorithm was first run for each library independently. Bedtools was used to identify clusters that were present in at least two out of three libraries in at least one condition (galls or roots). If these clusters were separated by a distance of less than 2 nucleotides, they were then merged (default parameter) and selected to build a reference set of clusters to perform counting and statistical analysis. From counting data, a DESeq statistical analysis was performed to identify clusters that were differentially expressed between gall and root conditions. Only clusters with a DicerCall (DC-clusters) and a minimum coverage of 2 rpmm in all replicates in at least one condition (galls or roots) were considered as biologically relevant

We sequenced small RNAs expressed in uninfected root inter-nodes and galls 7 and 14 dpi induced by the RKN *M. incognita*. Twelve small RNA libraries corresponding to three independent replicates of galls (G) at 7 and 14 dpi and the corresponding uninfected roots (R) were sequenced. The pipeline used for analysis of small RNAs is presented in Fig. 1. Roots galls are composed of nematode and plant tissue. The vast majority of the reads aligned with *A. thaliana* genome (90–95%) with only a small proportion (5–10%) of the reads aligned with *M. incognita* genome [40].

**Fig. 2:**
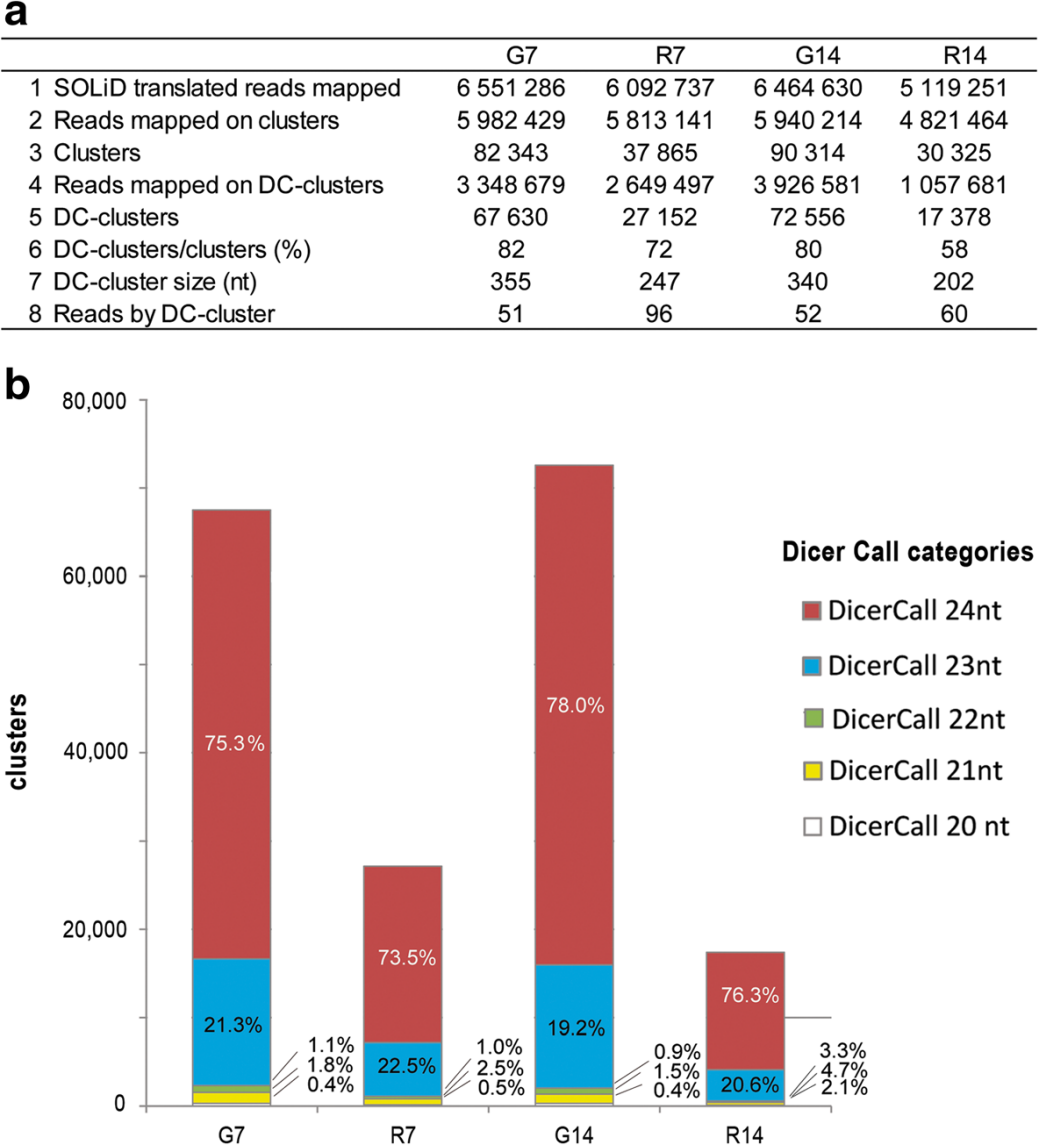
Characteristics of the clusters in galls (G) and uninfected roots (R) libraries at 7 and 14 dpi. **a** Average characteristics for the three libraries of each condition (G7, R7, G14 and R14): (1) translated SOLiD color spaced reads mapped on a *A. thaliana* genome; (2) reads mapped on the genomic regions associated with siRNA accumulation (clusters) on *A. thaliana* genome; (3) mean number of clusters per condition; (4) reads mapped on clusters with a Dicer Call (DC-clusters) i.e. when more than 80% of reads on the cluster have a size between 20 and 24 nt; (5) mean number and (6) proportion of DC-clusters; (7) DC-cluster mean size (nt) and (8) mean abundance of reads by DC-cluster. The data for each library are presented in Additional file 2: Table S1. **b** Stacked bar charts of the number of the various categories of DC-clusters (20, 21, 22, 23, 24 nt) for each condition. The proportions of each category are also presented

We predicted siRNA clusters, by annotation with ShortStack package [41] for each of the 12 libraries. Most of the sequenced reads mapped on *A. thaliana* genome are located within clusters (Fig. 2a). siRNAs result of the processing by Dicer of a long dsRNA precursor [42]. A DicerCall (DC) score is attributed by ShortStack to each cluster for which the majority of reads are between 20 and 24 nt in size, a characteristic typical of Dicer processing. A DC cutoff of 0.8 (meaning that at least 80% of the reads are between 20 and 24 nt long) was used to distinguish between non-DCL-derived and DCL-derived loci and to exclude all small RNAs corresponding to the degradation products of long RNAs (mRNAs, rRNAs, tRNAs) [43]. Most of the sequenced reads that are located within cluster on *A. thaliana* genome belong to DC-clusters (Fig. 2a, Additional file 1: Figure S1). Moreover, most of the clusters predicted were found to be DC-clusters: about 80% of the clusters in galls at 7 and 14 dpi, 72% in uninfected roots at 7 dpi and only 58% in uninfected roots at 14 dpi (Fig. 2a, Additional file 1: Figure S1). The lower proportion of DC-clusters in uninfected roots at 14 dpi principally reflects the effect of the R3–14 dpi library, which contained only 44% DC clusters (Additional file 2: Table S1). Only the R3–14 dpi library had a smaller number of reads mapping to DC-clusters (678,080) than the other libraries suggesting that the R3–14 dpi library may contain more degradation products than the other libraries. siRNA clusters produce a mixture of RNAs of different sizes. The DC score (21, 22, 23 or 24 nt) therefore corresponds to the most frequent size of reads mapped to the cluster and does not always reflect the diversity of reads within the cluster (Additional file 1: Figure S1). In all libraries, the majority of DC clusters (between 94 and 97%) had scores of 23 or 24 nt (Fig. 2). Only the DC clusters were retained for further analysis. As previously described [43], we assigned the predicted siRNA DC-clusters into two categories: 20–21-22 nt clusters corresponding to siRNAs and 23–24 nt clusters corresponding to hc-siRNAs.

About twice as many DC clusters were identified in galls as in uninfected roots, with a mean of 67,630 clusters at 7 dpi and 72,556 clusters at 14 dpi for galls, and a mean of 27,151 clusters at 7 dpi and 17,378 clusters at 14 dpi for uninfected roots (Fig. 2a). The mean size of uninfected root clusters was 247 nt at 7 dpi and 202 nt at 14 dpi, whereas the corresponding sizes in galls were 355 nt at 7 dpi and 340 nt at 14 dpi (Fig. 2a; Additional file 2: Table S1). The larger size of clusters in galls indicates that the over-representation of clusters in galls is not due to a prediction bias for size fragmentation. In addition to the much larger number of clusters in galls than in uninfected roots, the coverage of clusters was also clearly different at 7 dpi, with mean coverage rates of 51 and 96 reads per cluster in galls and roots, respectively. At 14 dpi, no strong difference was observed, with a mean coverage of 52 and 60 reads per cluster in galls and roots, respectively.

### Localization of siRNA clusters that are differentially expressed in galls

**Fig. 3:**
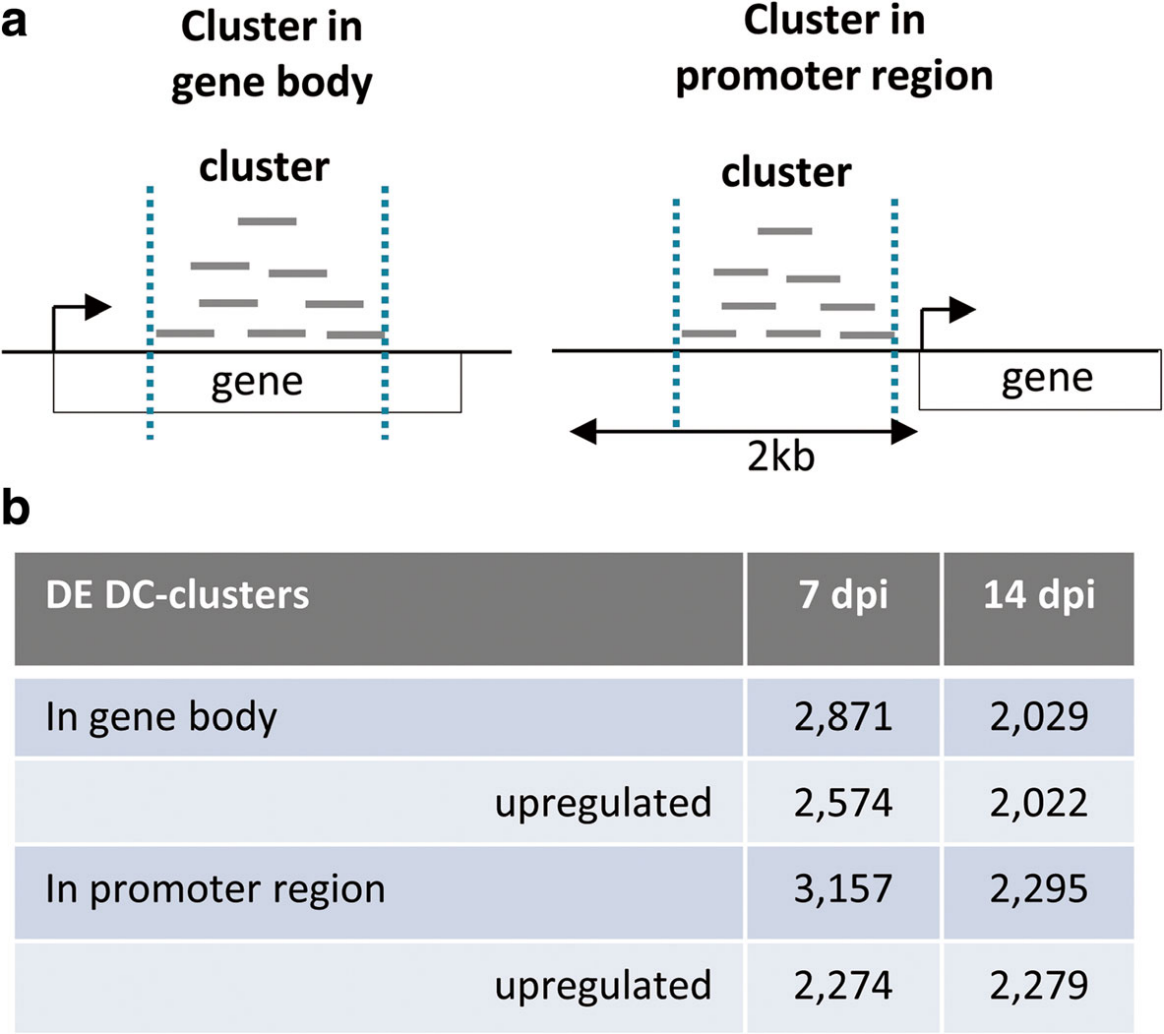
Different categories of DC-clusters according to their genomic location. **a** two classes of DC-clusters differentially expressed in galls vs roots were defined: clusters located within gene body or in 2 kb promoter region. **b** Table with the total number of clusters differentially expressed in galls in comparison to uninfected roots at 7 and 14 dpi according to their genomic location. The number of upregulated clusters is indicated

As siRNA clusters were predicted independently for each sample, the three libraries for each condition (gall or root) did not yield the same lists of predicted siRNA clusters (Additional file 2: Table S1). For the statistical analysis of our data, we constructed a reference set of predicted siRNA clusters with a strategy similar to that used for *Physcomitrella patens* [43]. For each time point, predicted *A. thaliana* siRNA clusters common to at least two of the three libraries in at least one condition (gall or root) were pooled in a reference set of predicted siRNA clusters (Fig. 1). This reference set comprised 86,264 and 92,578 siRNA clusters from gall and/or root libraries at 7 and 14 dpi, respectively. We compared the expression levels of these predicted siRNA clusters between galls and roots at each time point, by DESeq statistical analysis [44] with an adjusted *P*-value (p_adj_) < 0.05 considered significant. Only differentially expressed (DE) clusters supported by a DC score and displaying significant levels of expression (at least 2 reads per million mapped reads (RPMM)) in the three libraries, for at least one condition (gall or root) were considered to be robust predicted siRNA clusters and were retained for further analysis (Additional file 3: Table S2). An analysis of the genomic positions of predicted differentially expressed siRNA clusters showed that, at 7 dpi, 2871 and 3157 clusters in galls were located within the body of the gene and in the promoter region, respectively (Fig. 3; Additional file 4: Table S3). We found that 2029 and 2295 of the clusters differentially expressed in galls at 14 dpi were located within gene sequence and putative promoter regions, respectively (Fig. 3; Additional file 4: Table S3). The numbers of clusters in putative promoter regions and within the body of genes were therefore similar. Most of these clusters were covered by reads in both gall and root samples. Overall, 275 and 809 clusters upstream from genes at 7 and 14 dpi, respectively, and 328 and 653 clusters within genes at 7 and 14 dpi, respectively, were covered exclusively by reads in a single condition, either galls or roots (Additional file 5: Table S4). Most of the predicted DE clusters were found to be upregulated in galls (Fig. 3; Additional file 4: Table S3). At 7 dpi, the clusters upregulated in galls accounted for 72.0% of the clusters located in promoter regions and 89.7% of those located within the body of the gene. At 14 dpi, the clusters upregulated in galls accounted for 99.3% of the clusters located in promoter regions and 99.7% of those located within the body of the gene. Only two clusters were among the 20 differentially expressed clusters with the highest fold change in expression, both at 7 and 14 dpi: one of these clusters is located within a gene encoding a lecithin cholesterol acyltransferase (AT3G44830), and the other is located upstream from a gene encoding a homeodomain-like protein (AT2G13960).

**Fig. 4:**
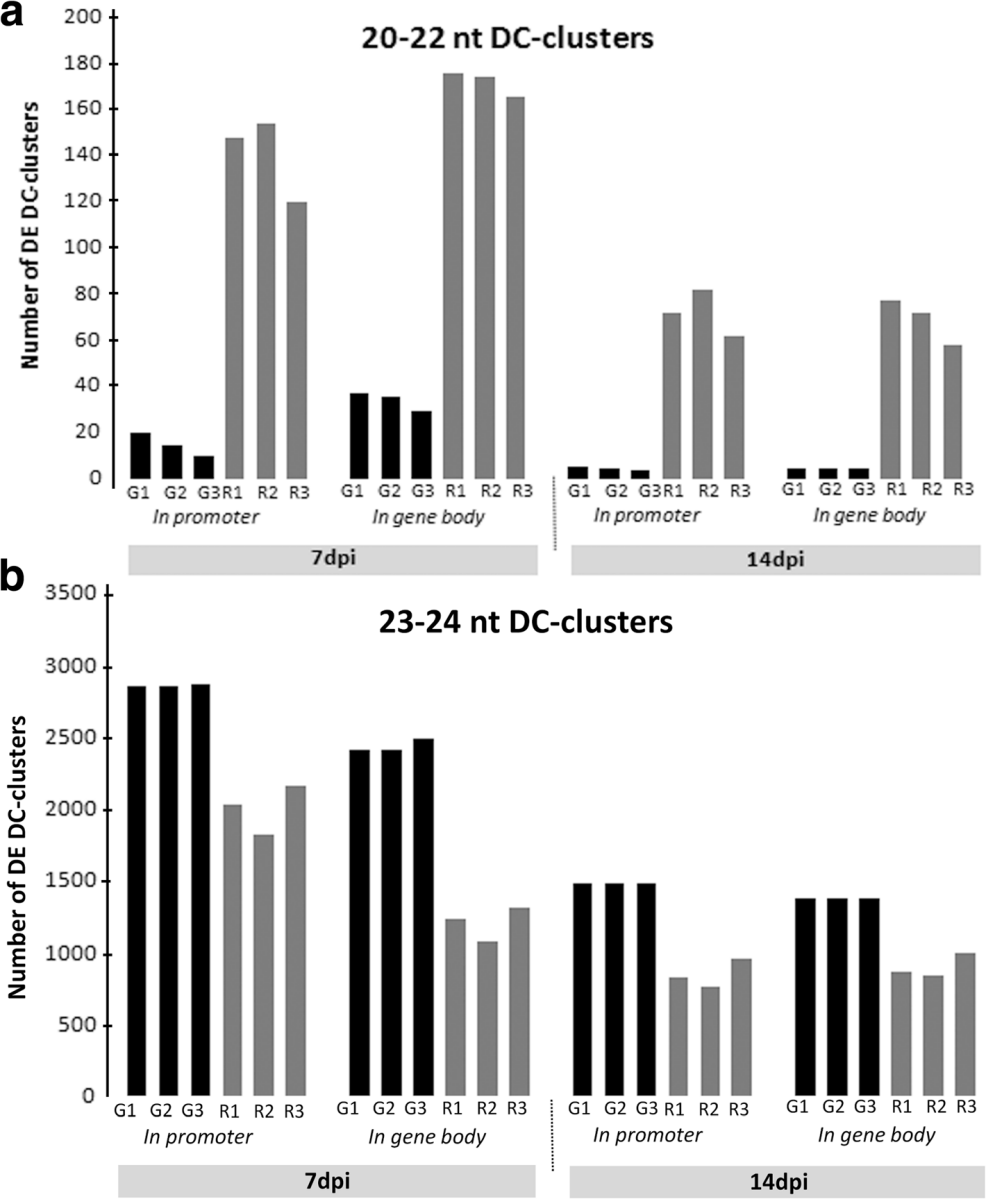
Differentially expressed DC-clusters in galls and roots according to their DC size and their genomic location. Number of differentially expressed (DE) DC-clusters in the twelve galls (G) and roots (R) libraries at 7 and 14 dpi according to their DC size and their genomic location: within gene body or promoter regions. **a** Number of clusters of the 23–24 nt category. **b** Number of clusters of the 20–22 nt category

The number of clusters was always larger in galls than in uninfected roots (Additional file 6: Table S5) and 23–24 nt was the main DC category of DE clusters regardless of the type of sample (galls or uninfected roots) or the genomic location (in promoter or in gene body) in comparison to the 20–22 nt DC category (Additional file 6: Table S5). However, these two DC categories clearly differed between galls and roots: the number of 23–24 nt clusters in galls was always larger than in uninfected roots, but the opposite pattern was observed for the 20–22 nt category, for which the number of clusters in uninfected roots was always greater than in galls (Fig. 4 and Additional file 6: Table S5). The over-representation differentially expressed clusters in galls appeared therefore to be specific to the 23–24 nt category. This size corresponds to the hc-siRNAs that are known to repress gene expression by targeting transposable elements located in their promoter region and by inducing RdDM. We therefore focused further analyses on the differentially expressed clusters located in putative promoter regions by i) strengthening cluster bioinformatic predictions with transcriptomic data and ii) investigating the presence of transposable elements or repeats derived from transposable elements within these biologically relevant clusters.

### Analysis of differentially expressed clusters of hc-siRNAs and their putative targeted genes in galls

We restricted our analysis to biologically relevant genes, by focusing on predicted DE siRNA clusters located in the putative promoters of genes differentially expressed in galls with inversely correlated expression profiles. The expression of genes located within 2 kb downstream from the siRNA clusters was investigated within DNA chips data listed in NEMATIC [45]. Only a small fraction of the predicted siRNAs targeted genes differentially expressed in galls (Additional file 3: Table S2). At 7 dpi, 118 genes covered by 129 differentially expressed siRNA clusters and located within the putative promoter region were differentially expressed in galls and the expression profiles of 59 of these genes were inversely correlated with those of the corresponding siRNA clusters, with 37 genes repressed and 22 upregulated in galls (Table 1; Additional file 7: Table S6). Of these 59 DE genes, 14 genes (23.7%) were specifically differentially expressed at 7 dpi and 31 genes (52.5%) were differentially expressed at 7, 14 and 21 dpi (Table 1). Twenty-nine of these 31 genes had similar patterns of expression at these three time points: 20 were downregulated and nine were upregulated in galls, throughout gall development.

**Table 1:** Top ten most upregulated and downregulated siRNA DC-clusters in galls compared to uninfected roots at 7 dpi

DC-clusters			Downstream genes			
Cluster name	variation G/R 7dpi	gene name	Gene description (TAIR)	Log2 G/R		
				7 dpi	14 dpi	21 dpi
chr4:5373687–5,374,187	up	*At4g08455*	BTB/POZ domain-containing protein	−0.9	No	− 0.9
chr1:17450567–17,451,109	up	*At1g47530*	MATE efflux family protein	−0.8	No	−1.0
chr3:10387344–10,387,729	up	*At3g27970*	Exonuclease family protein	−1.1	No	−0.9
chr2:11040438–11,040,725	up	*At2g25900*	*A. thaliana* TANDEM ZINC FINGER PROTEIN 1 (ATCTH)	−0.8	−0.8	−1.4
chr3:9814548–9,815,346	up	*At3g26720*	glycosyl hydrolase family 38 protein	−1.0	No	No
chr3:22340217–22,340,552	up	*At3g60450*	Phosphoglycerate mutase family protein	−1.2	−1.4	− 1.5
chr3:15356722–15,357,007	up	*At3g43430*	RING/U-box superfamily protein (zinc finger)	−0.7	−0.9	No
chr1:25745346–25,745,737	up	*At1g68570*	membrane localized GA transporter (ATNPF3.1)	−1.2	−1.5	−1.3
chr1:24996663–24,997,231	up	*At1g66970*	GLYCEROPHOSPHODIESTER PHOSPHODIESTERASE (GDPD) LIKE 1	−0.9	−1.5	−0.9
chr2:1681688–1,682,004	up	*At2g04795*	unknown protein	−1.2	No	No
chr3:10284167–10,284,467	down	*At3g27740*	carbamoyl phosphate synthetase (CPS) small subunit (carA)	0.7	No	No
chr1:26139675–26,140,148	down	*At1g69530*	Expansin A1	1.3	2.9	3.0
chr1:10506341–10,507,511	down	*At1g29980*	choice-of-anchor C domain protein	1.1	1.9	1.4
chr4:2442915–2,443,871	down	*At4g04830*	methionine sulfoxide reductase B5	1.9	No	No
chr3:22214692–22,214,777	down	*At3g60140*	protein similar to beta-glucosidase	0.7	−2.1	−1.2
chr2:1281392–1,281,621	down	*At2g04030*	chloroplast-targeted 90-kDa heat shock protein (CR88)	1.0	0.9	No
chr1:3322776–3,323,550	down	*At1g10140*	unknown protein	0.9	No	0.7
chr5:19447678–19,447,954	down	*At5g48000*	Member of the CYP708A family of cytochrome P450 enzymes (CYP708A2)	0.8	No	No
chr2:14572190–14,572,294	down	*At2g34590*	transketolase family protein	0.8	1.1	0.7

These DC-clusters were all shared by galls and roots and located in promoter regions and with expression patterns inversely correlated with those of the downstream differentially expressed genes. The upregulation (up) or downregulation (down) of the cluster in galls (G) compared to uninfected roots (R) at 7 dpi (variation G/R 7 dpi), the AGI gene name, the description of the encoded protein from TAIR and the log2 values of galls/roots at 7, 14 and 21 dpi obtained from microarrays [32, 45] were indicated

At 14 dpi, 116 genes covered by 129 DE siRNA clusters in their promoter regions were differentially expressed in galls, and the expression profiles of 69 of these genes were inversely correlated with those of the siRNA clusters; all of these genes were repressed in galls (Table 2; Additional file 8: Table S7). Fifteen (21.7%) of the 69 DE genes with expression patterns inversely correlated with that of the DE clusters in their putative promoter regions were differentially expressed specifically at 14 dpi, 16 genes (23.2%) were differentially expressed at 7, 14 and 21 dpi. 34 genes (49.3%) were differentially expressed at 14 and 21 dpi, but not at 7 dpi (Additional file 8: Table S7).

**Table 2:** Top ten most upregulated siRNA DC-clusters in galls compared to uninfected roots at 14 dpi

DC-clusters			Downstream genes			
Cluster name	variation G/R 14 dpi	gene name	Gene description (TAIR)	Log2 G/R		
				7 dpi	14 dpi	21 dpi
chr1:25745518–25,745,718	up	*At1g68570*	Membrane localized GA transporter (NPF3.1)	−1.2	− 1.5	−1.3
chr1:9412541–9,412,703	up	*At1g27100*	Actin crosslinking protein	−1.2	−0.9	−1.0
chr2:18703172–18,703,490	up	*At2g45380*	Myeloid leukemia factor	No	−1.1	−1.6
chr4:1063076–1,063,502	up	*At4g02410*	Concanavalin A-like lectin protein kinase family	No	−0.7	− 0.6
chr5:16944206–16,944,689	up	*At5g42380*	Calmodulin like 37 (CML37)	−1.4	−2.0	−2.4
chr3:17722355–17,722,693	up	*At3g48020*	Hypothetical protein	No	−0.8	−0.8
chr5:14896137–14,896,289	up	*At5g37500*	Gated outwardly-rectifying K+ channel	No	−0.7	− 0.6
chr4:8677540–8,679,013	up	*At4g15230*	Pleiotropic drug resistance 2 (PDR2); ATPase	No	−1.0	−0.7
chr1:23262107–23,262,634	up	*At1g62810*	Copper amineoxidase 1 (CuAO1)	No	−1.3	No
chr4:7855461–7,857,189	up	*At4g13510*	Plasma membrane localized ammonium transporter (AMT1;1)	−0.7	−1.0	−0.6

These DC-clusters were all shared by galls and roots and located in promoter regions and with expression patterns inversely correlated with those of the downstream differentially expressed genes. The upregulation (up) of the cluster in galls (G) compared to uninfected roots (R) at 14 dpi (variation G/R 14 dpi), the AGI gene name, the description of the encoded protein from TAIR and the log2 values of galls/roots at 7, 14 and 21 dpi obtained from microarrays [32, 45] were indicated

A comparison of the results presented in Additional file 7: Table S6 and Additional file 8: Table S7 identified 13 genes as differentially expressed with expression patterns inversely correlated with those of the DE siRNA-clusters targeting their putative promoters at 7 and 14 dpi (Additional file 9: Table S8). These genes included several with molecular functions relating to catalytic activity (2-oxoglutarate (2OG) and Fe(II)-dependent oxygenase, choline kinase 3, Phosphoglycerate mutase, beta-glucosidase and NADP-dependent malic enzyme 2), or encoding DNA-binding proteins (protein-coding NAC domain-containing protein 58).

### Colocalisation of hc-siRNAs clusters and repeats

**Fig. 5:**
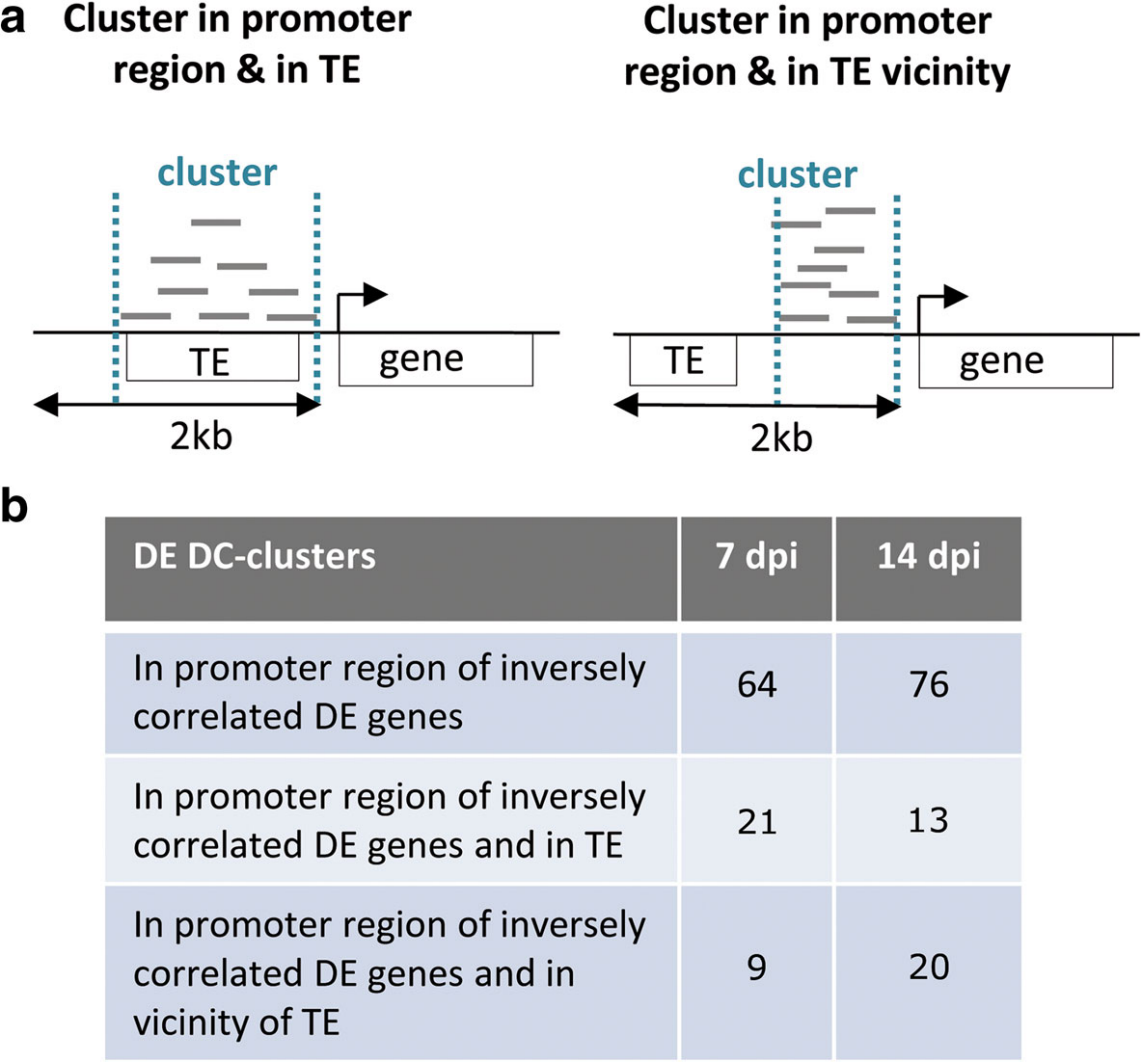
Location of differentially expressed DC-clusters within promoter regions regarding location of transposable elements. **a** Among the differentially expressed (DE) siRNA clusters located in the promoter of genes differentially expressed in galls with an inversely related expression profile, clusters located within TE (left panel) or in the vicinity of TE (right panel) are the best candidates to regulate gene expression in galls by RdDM pathway. **b** Table with the number of clusters differentially expressed in galls in comparison to uninfected roots according to their location compared to transposable elements: within TE or in the vicinity of TE

Genes targeted by hc-siRNA and RdDM have TEs, or repeats derived from TEs, within their promoter sequences. These sequences are targeted by hc-siRNA, to induce TGS of this region through the induction of RdDM. We investigated the presence of transposon-derived repeats within or in the vicinity of the differentially expressed siRNA clusters. We retrieved the 2 kb upstream from the 5’UTR of DE genes with siRNA clusters in their promoter region and inversely correlated pattern of expression for the gene and the siRNA cluster and investigated the presence of repeats within or close to the cluster by comparing these sequences to Repbase sequences with CENSOR [46]. At 7 dpi, 21 of the 64 clusters differentially expressed in galls, located in promoter regions and with expression patterns inversely correlated with those of the associated DE genes had sequences displaying identity to the *A. thaliana* TE (Fig. 5; Table 3). At 14 dpi, 13 of the 76 clusters differentially expressed in galls, located in promoter regions and displaying expression patterns inversely correlated with those of the associated DE genes displayed had sequences displaying identity to the *A. thaliana* TE (Fig. 5; Table 4). The other DC-clusters displayed no direct sequence identity but were located in the vicinity of repeats. Eight genes differentially expressed at 7 dpi and 17 genes differentially expressed at 14 dpi had siRNA clusters and sequences displaying identity to the sequences of transposable elements in the promoter region, although the transposable elements and siRNA clusters did not overlap (Fig. 5; Table 5; Table 6). These genes correspond to 9 DE clusters at 7 dpi and 20 at 14 dpi that have sequence identity with TE in their vicinity. All together, 46% of the clusters differentially expressed in galls at 7 dpi and 39% of the clusters differentially expressed in galls at 14 dpi display sequence identity with *A. thaliana* TE or are located in vicinity of sequence homologous to TE.

**Table 3:** siRNA DC-clusters differentially expressed in galls at 7dpi with sequence identity to Arabidopsis transposable elements

DC-clusters			Downstream genes			TE
Cluster name	variation G/R 7dpi	Gene name	Gene description (TAIR)	Log2 G/R 7 dpi	Similarity	TE name
chr1:10506341–10,507,511	down	*At1g29980*	Protein of unknown function	1.1	0.8863	BOMZH2
					0.9127	BOMZH2
chr1:11721631–11,721,864	up	*At1g32450*	Transmembrane nitrate transporter	−1.0	0.9526	ATREP4
chr1:13544313–13,545,488	down	*At1g36180*	acetyl-CoA carboxylase 2 acetyl-CoA carboxylase 2 (ACC2)	0.8	0.8685	ATHATN1
					0.7852	VANDAL16
chr1:9412110–9,412,695	up	*At1g27100*	Actin cross-linking protein	−1.2	0.8485	AtSB2
chr1:9479725–9,480,634	up	*At1g27290*	unknown protein	−1.1	0.8485	ATCOPIA3I
					1.0000	ATCOPIA3LTR
chr2:1281392–1,281,621	down	*At2g04030*	Chaperone protein htpG family	1.0	0.9291	ATREP13
chr2:14571706–14,572,100	down	*At2g34590*	Transketolase family protein	0.8	0.9466	ATREP1
chr2:14572190–14,572,294	down	*At2g34590*			0.9505	ATREP1
chr2:15595033–15,595,856	down	*At2g37120*	S1FA-like DNA-binding protein	0.8	0.9756	ATMU7
					0.9863	ATMU7
chr2:8939001–8,939,617	down	*At2g20750*	expansin B1	0.7	0.7231	BRODYAGA1A
chr3:10284167–10,284,467	down	*At3g27740*	carbamoyl phosphate synthetase A	0.7	0.7131	TSCL
chr3:22214692–22,214,777	down	*At3g60140*	Glycosyl hydrolase superfamily protein	0.7	0.8537	AtSB2
chr4:2442915–2,443,871	down	*At4g04830*	methionine sulfoxide reductase B5	1.9	0.9746	VANDAL3
chr4:2444411–2,445,805	down	*At4g04830*			0.9860	VANDAL3
chr4:5373687–5,374,187	up	*At4g08455*	BTB/POZ domain-containing protein	−0.9	0.6833	ATLINE1 6
chr4:6489475–6,490,728	up	*At4g10500*	2-oxoglutarate (2OG) and Fe(II)-dependent oxygenase superfamily protein	−0.8	0.7176 0.8434	ATHAT8 ATHATN4
chr4:9035216–9,035,789	down	*At4g15930*	Dynein light chain type 1 family (DIN2)	1.4	0.9623	ATTIRTA1
chr5:16211264–16,212,381	down	*At5g40480*	embryo defective 3012	0.7	0.7356	ATREP13
chr5:16944212–16,945,214	up	*At5g42380*	calmodulin like 37	−1.4	0.7117	TAG1
chr5:19447678–19,447,954	down	*At5g48000*	cytochrome P450, family 708, subfamily A	0.8	0.9667	ATRAN
chr5:3750834–3,753,083	up	*At5g11670*	NADP-malic enzyme 2	−1.4	0.8554	ATHATN4

siRNA DC-clusters differentially expressed in galls compared to uninfected roots at 7 dpi located in promoter regions and with expression patterns inversely correlated with those of the associated differentially expressed genes and displaying sequence identity to *A. thaliana* transposable elements (TE). The upregulation (up) or down regulation (down) of the cluster in galls (G) compared to uninfected roots (R) at 7 dpi (variation G/R 7 dpi), the AGI gene name, the description of the encoded protein from TAIR and the log2 values of galls/roots at 7 dpi obtained from microarrays [32, 45] were indicated

**Table 4:** siRNA DC-clusters differentially expressed in galls at 14dpi with sequence identity to Arabidopsis transposable elements

DC-clusters			Downstream genes				TE
Cluster name	variation	Gene name	Gene description (TAIR)	Log2 G/R		Similarity	TE name
	G/R 14 dpi			7 dpi	14 dpi		
chr1:17018600–17,021,142	up	*At1g45015*	MD-2-related lipid recognition domain-containing protein	No	−1.6	0.7642	TNAT2A
chr2:14492683–14,493,021	up	*At2g34350*	Nodulin-like / Major Facilitator Superfamily	No	−0.7	0.8050	ATTIRX1A
chr2:18083599–18,083,939	up	*At2g43590*	Chitinase family	No	−1.1	0.7284	ATTIRX1B
chr2:5923051–5,923,711	up	*At2g14080*	Disease resistance protein (TIR-NBS-LRR class)	No	−0.7	0.8189	ATLINE1A
chr2:5924097–5,924,304	up					0.6909	ATCOPIA95 I
chr3:10719116–10,720,702	up	*At3g28600*	P-loop containing nucleoside triphosphate hydrolases	No	−1.0	0.7458	Sadhu7–2
chr3:11195491–11,195,822	up	*At3g29250*	NAD(P)-binding Rossmann-fold superfamily protein	No	−0.9	0.7312	ATHILA4D LTR
chr3:22215861–22,216,213	up	*At3g60140*	Protein similar to beta-glucosidase and is a member of glycoside hydrolase family 1 (DIN2, SRG2)	0.7	−2.1	0.9794	ATREP13
chr4:6489725–6,490,158	up	*At4g10500*	Oxidoreductase, 2OG-Fe(II) oxygenase family protein	−0.8	2.3	0.7176	ATHATN3
						0.7826	ATHAT8
chr4:6490209–6,490,725	up					0.7794	ATHATN4
						0.8434	ATHATN4
chr5:20165643–20,165,928	up	*At5g49660*	Xylem intermixed with phloem 1	No	−1.2	0.7474	ATHATN3
chr5:3750834–3,753,121	up	*At5g11670*	Malic enzyme (NADP-ME2)	−1.4	−1.0	0.8554	ATHATN4
chr5:5716875–5,717,705	up	*At5g17340*	Putative membrane lipoprotein	No	−2.1	0.7153	AtSB4

siRNA DC-clusters differentially expressed in galls compared to uninfected roots at 14 dpi located in promoter regions and with expression patterns inversely correlated with those of the associated differentially expressed genes and with sequence identity to *A. thaliana* TE. The upregulation (up) of the cluster in galls (G) compared to uninfected roots (R) at 7 dpi (variation G/R 7 dpi), the AGI gene name, the description of the encoded protein from TAIR and the log2 values of galls/roots at 7 and 14 dpi obtained from microarrays [32, 45] were indicated

**Table 5:** siRNA DC-clusters differentially expressed in galls at 7dpi located in the vicinity of Arabidopsis transposable elements

DC-cluster name	Gene name	Gene description	TE similarity	TE name
chr1:17450567–17,451,109	*At1g47530*	MATE efflux family protein	1.3333	ARNOLDY1
chr1:26139675–26,140,148 chr1:26140251–26,140,500	*At1g69530*	Expansin A1	2.3000	RP1_AT
chr2:1681688–1,682,004	*At2g04795*	hypothetical protein	1.6000	ATHPOGON3
			1.6250	ATHPOGON2
chr3:3587400–3,587,740	*At3g11410*	Phosphatase 2C	1.9000	ATCOPIA75LTR
			1.2333	ATHATN4
chr3:9814548–9,815,346	*At3g26720*	Glycosyl hydrolase family 38 protein	2.3333	ATREP10B
			1.7273	ATHPOGON3
chr3:20629820–20,629,940	*At3g55610*	delta 1-pyrroline-5-carboxylate synthetase B	2.8333	RP1 AT
chr4:5791975–5,792,114	*At4g09030*	arabinogalactan protein (AGP10).	1.1818	BOMZH1
chr2:2232130–2,232,414	*At2g05840*	20S proteasome subunit PAA2	1.7619	SIMPLEGUY1
			1.8333	TAG2
			1.6047	ATMUNX1
			1.4730	SIMPLEGUY1

siRNA DC-clusters differentially expressed in galls at 7 dpi located in promoter regions, with expression patterns inversely correlated with those of the associated differentially expressed genes and located in the vicinity of *A. thaliana* TE. The AGI gene name, the description of the encoded protein from TAIR, the TE similarity and TE name were indicated

**Table 6:** siRNA DC-clusters differentially expressed in galls at 14dpi located in the vicinity of Arabidopsis transposable elements

DC-cluster name	Gene name Gene description		TE similarity	TE name
chr1:8383930–8,384,530	*At1g23710*	hypothetical protein	0.7606	ATHATN9
chr1:9412105–9,412,396 chr1:9412541–9,412,703	*At1g27100*	Actin cross-linking protein	0.8485	AtSB2
chr1:11737691–11,737,819	*At1g32460*	hypothetical protein	0.9656	ATREP3
chr1:12890229–12,890,337	*At1g35190*	2-oxoglutarate (2OG) and Fe(II)-dependent oxygenase	0.7529	ATLINE1 5
			0.8714	ATHATN2
chr1:24483137–24,483,294	*At1g65820*	microsomal glutathione s-transferase	0.9008	ATHPOGON3
chr2:743734–743,891	*At2g02680*	Cysteine/Histidine-rich C1 domain family	0.8144	TNAT1A
			0.8421	DT1
			0.7864	TNAT1A
chr2:7123544–7,123,869	*At2g16430*	purple acid phosphatase 10	0.7983	ATMUNX1
chr2:9979300–9,979,500	*At2g23430*	cyclin-dependent kinase inhibitor protein (KRP1)	0.8657	ATTIR16T3A
chr2:10139020–10,139,268	*At2g23810*	Tetraspanin Tet8	0.7800	BRODYAGA1A
chr2:18703172–18,703,490	*At2g45380*	myeloid leukemia factor	0.9818	ATLINEIII
chr3:9964887–9,966,036	*At3g27020*	metal-nicotianamine transporter YSL6	0.7614	ATREP7
chr3:11031230–11,031,528 chr3:11030736–11,031,214	*At3g29034*	transmembrane protein	0.6516	ATCOPIN_LTR
chr4:6144430–6,145,149	*At4g09750*	NAD(P)-binding Rossmann-fold superfamily	0.6691	ATLINE2
chr4:6312052–6,312,340	*At4g10110*	RNA recognition motif (RRM)-containing protein	0.7590	ATMUN1
			0.7882	ATLINE1A
			0.8201	ATREP1
chr5:8671661–8,672,311	*At5g25140*	Cytochrome P450	0.8462	ATREP2
chr5:16944206–16,944,689	*At5g42380*	calmodulin like 37	0.7117	TAG1
chr5:25936470–25,936,892	*At5g64900*	Putative 92-aa protein that is the precursor of AtPep1	0.8824	ATREP6
chr5:25935269–25,935,884			0.8854	ATREP6

List siRNA DC-clusters differentially expressed in galls at 14 dpi located in promoter regions, with expression patterns inversely correlated with those of the associated differentially expressed genes and located in the vicinity of *A. thaliana* TE. The AGI gene name, the description of the encoded protein from TAIR, the TE similarity and TE name were indicated

### Differentially expressed genes with siRNA clusters colocalised with repeats in their promoter regions overlap with genes differentially expressed in the syncytium induced by cyst nematode with methylation-associated TE profiles

Cyst nematode induce the formation of hypermetabolic multinucleate feeding site, named syncytium, that results from the induction of an initial syncytial cell within the root parenchyma that then integrates several hundred of the surrounding cells through cell wall dissolution [47, 48]. Therefore the feeding sites induced by CN and RKN differ by their biogenesis but have similar phenotype and share some molecular pathways [49]. We compared the list of DE genes/DE siRNA clusters in galls displaying inversely correlated patterns of expression and with repeats in the gene promoter to the list of the 526 differentially methylated TE-associated genes corresponding to genes differentially expressed in syncytia. We only identified seven genes as differentially methylated TE-associated genes that were differentially expressed in syncytia [38] and targeted by differentially expressed siRNA clusters in GCs at 7 or 14 dpi (Table 7). These genes were differentially expressed in galls and syncytia, with similar expression patterns, had repeats/TEs in their putative promoter regions, predicted siRNA clusters differentially expressed in galls with inversely correlated patterns of expression to the gene and were differentially methylated in syncytia and uninfected roots. These seven robust candidates for regulation by RdDM during the plant-CN/RKN interactions encode a receptor protein for CEP1 peptide (AT5G49660) involved in the maintenance organization of cell files or cell morphology in conductive elements, a xylem nitrate transporter (AT1G32450), a protein involved in oxidative stress (methionine sulfoxide reductase B5, AT4G04830), a 20S proteasome subunit (AT2G05840), a tetraspanin (AT2G23810), a protein similar to beta-glucosidase (AT3G60140) and a protein of unknown function (embryo defective 3012, AT5G40480).

**Table 7:** siRNA DC-clusters differentially expressed in galls at 7 and/or 14dpi colocalised with differentially methylated regions in cyst nematode feeding sites

Gene name	Log2 G/R		Log2 Sync/R	Gene description	Clusters	CENSOR
	7 dpi	14 dpi	5 + 15dpi			
7 dpi DE DC clusters						
At1g32450	−1.0	−1.0	−3.9	Transmembrane nitrate transporter	chr1:11721631–11,721,864	ATREP4
At2g05840	0.8	No	0.9	20S proteasome subunit	ch r2:2232130–2,232,414	ATMUNX1& SIMPLEGUY1 & TAG1
At3g60140	0.7	−2.1	2.7	protein similar to beta-glucosidase	chr3:22214692–22,214,777	AtSB2
At4g04830	1.9	No	0.5	methionine sulfoxide reductase B5	ch r4:2442915–2,443,871 ch r4:2444411–2,445,805	VANDAL3 VANDAL3
At5g40480	0.7	0.7	1.3	Embryo defective 3012	chr5:16211264–16,212,381	ATREP13
14 dpi DE DC clusters						
At2g23810	−1.0	−0.9	−3.3	Tetraspanin 8	chr2:10139020–10,139,268	BRODYAGA1A
At5g49660	No	−1.2	−1.0	C-terminally encoded peptide receptor 1	ch r5:20165643–20,165,928	ATHATN3

siRNA DC-clusters differentially expressed (DE) in galls at 14dpi located in the vicinity of Arabidopsis transposable elements. List of siRNA DC-clusters differentially expressed in galls at 7 dpi and/or 14 dpi located in promoter region, with expression patterns inversely correlated with those of the associated differentially expressed genes and that colocalise with differentially methylated regions in cyst nematode feeding sites. The AGI gene name, the description of the encoded protein from TAIR and the log2 values of galls/roots (G/R) at 7 and 14 dpi obtained from microarrays [32, 45] and the log2 values of syncytia/roots (Sync/R) at 5 and 15 dpi obtained from [38] were indicated

## Discussion

The classification of siRNAs is complex, and their study requires a new analysis at genome level for each biological condition analyzed. For this genome-wide analysis, we used the ShortStack bioinformatics tool [41], which i) was developed for plant genomes, ii) predicts de novo areas of the genome in which small RNAs accumulate, named “clusters” and (iii) carries out statistical analyses of the read counts corresponding to these clusters, comparing the gall to root conditions. This algorithm therefore performs clustering analyses rather than comparing the expression levels of single short sequences. In addition to predicting clusters generating siRNAs, ShortStack also provides information about the small-RNA population of each cluster, including the probability that this cluster results from maturation by a Dicer protein (DicerCall), size distribution, position on the strand, and the most highly represented sequence at the locus. We identified a large number of siRNA clusters in galls and uninfected roots, with the number of clusters predicted in uninfected root libraries much lower than that in galls. We investigated whether this larger number of clusters in the galls was due to the simple fragmentation of clusters into smaller clusters. The larger size of the clusters in galls was not consistent with such a prediction bias. The imbalance for 23–24 nt sequences seems to be characteristic of galls and has already been reported for galls collected at 3 dpi [36]. DESeq statistical analysis identified clusters differentially regulated between galls and uninfected roots. We increased the robustness of our data and the stringency of our analysis by selecting only differentially expressed clusters for which prediction was based on i) reproducible results for the various libraries, ii) a significant level of expression and iii) a high probability of resulting from Dicer cleavage.

The hc-siRNAs associated with TGS are mostly targeted to promoter regions, although some examples of hc-siRNAs targeting the body of the gene have been reported [7, 50]. For the study of hc-siRNAs, we therefore chose to focus on siRNA clusters targeting putative promoter regions. For the selection of clusters with regulatory action supported by biological evidence, we restricted our study to differentially expressed clusters with a DicerCall of 23–24 nt, covering promoter regions or genes differentially expressed in galls in DNA chip analysis and displaying a pattern of expression inversely correlated with that of the corresponding clusters. However, this strategy was highly restrictive, because only 2.7% of the genes with a cluster in their promoter region displayed transcriptomic regulation in NEMATIC DNA chip data. Similarly, only 5.6% of the DE siRNA clusters target a promoter region of a gene that is differentially expressed in galls at 7 and/or 14 dpi. The NEMATIC data were extracted from transcriptomic analyses performed with microarrays [45]. Microarrays are less sensitive and less exhaustive than new sequencing technologies [51]. New high-throughput sequencing-based analyses of gall transcriptomes are, therefore, required, to obtain a more complete view of the transcriptional reprogramming occurring in galls. The proportions of upregulated and downregulated siRNA clusters with inversely correlated expression differed between 7 and 14 dpi. At 7 dpi, 58 clusters (65.5%) were upregulated in galls, whereas, at 14 dpi, 100% of DE clusters were upregulated, suggesting a progressive increase in the number of clusters upregulated in galls. An analysis of the expression profiles of DE genes targeted by siRNA clusters in their putative promoters with an inversely correlated expression profile throughout gall development studied in [32] at 7, 14 and 21 dpi showed that most of the genes targeted by siRNA clusters at 7 dpi were differentially expressed at 7, 14 and 21 dpi, whereas most of the genes targeted by siRNA clusters at 14 dpi are differentially expressed at 14 and 21 dpi, but not at 7 dpi. However, these differences do not suggest any hypotheses concerning the action of siRNA, because these proportions are consistent with the general transcriptomic data obtained over the entire period of gall development [32]. Finally, TEs or sequences displaying identity to repeats were found in the putative promoter region of 40% (at 7 dpi) and 50.7% (at 14 dpi) of the DE genes displaying an inverse correlation of expression with the siRNA clusters in the promoter. These genes are good candidates for regulation by hc-siRNA during gall formation.

hc-siRNAs control transposons and gene expression by inducing DNA methylation. We compared the list of hc-siRNA clusters differentially expressed in galls and supported by biological expression data with the genomic regions that were identified as differentially methylated in feeding sites in *A. thaliana* roots induced by the cyst nematode *H. schachtii* [38]. The role of siRNAs in plant- CN nematode interactions was first highlighted by a significant lower rate of infection with *H. schachtii* in the *A. thaliana* mutants *dcl2–1* [37]. We identified five genes at 7 dpi and two genes at 14 dpi with i) promoter regions displaying some sequence identity to transposable elements ii) differential expression in galls and an inverse correlation of expression with DE siRNA clusters located in the promoter region of the genes concerned and iii) promoter regions differentially methylated in CN feeding sites. These genes are, therefore, robust candidates for regulation by hc-siRNA, through RdDM, during the development of feeding sites induced by plant-parasitic nematodes. Recently, two Arabidopsis mutants rdr2-rdr6 and dcl2/dcl3/dcl4 deprived of key factors for RdDM were shown to have lower rates of infection with RKN highlighting the importance of siRNAs and RdDM in Arabidopsis-Meloidogyne interaction [52].

## Conclusions

In this work we provide the first analysis of siRNA clusters expressed in root galls induced by the RKN *M. incognita*. We identified several siRNA clusters that are candidates to modify expression of plant genes in response to RKN infection and these regulations are supported by transcriptomic expression data. These candidates should now be investigated in functional analyses, to confirm i) their regulation by RdDM and ii) their role in plant-nematode interactions. siRNAs will constitute a new field of investigation in studies of the molecular mechanisms underlying plant responses to parasitic nematodes.

## Methods

### Biological material, growth conditions and nematode inoculation

Seeds of *A. thaliana* (ecotype Wassilewskija) were surface-sterilised and sown on Murashige and Skoog (Duchefa) medium agar plates (0.5 x MS, 1% sucrose, 0.8% agar, pH 6.4). Plates were kept at 4 °C for two days, and then transferred to a growth chamber (20 °C with 8 h light and 16 h darkness). *M. incognita* strain “Morelos” was multiplied on tomato plants in a growth chamber (25 °C, 16 h light and 8 h darkness). For in vitro nematode infection, J2 larvae were surface-sterilised with HgCl2 (0.01%) and streptomycin (0.7%) as described elsewhere [53]. We inoculated 25-day-old seedlings grown in vitro individually with 200 sterilised J2 s each, resuspended in Phytagel. Seven and 14 dpi, galls were dissected from the infected roots by hand. We also dissected internodes from uninfected roots (without apical and lateral root meristems) with the same age than infected roots for use as a negative control. Samples were immediately frozen in liquid nitrogen and stored at − 80 °C. Three independent biological replicates were established for each set of conditions.

### Construction and sequencing of small RNA libraries

Total RNA, including small RNAs (less than 200 nt long), was isolated from galls or uninfected roots at 7 and 14 dpi. Approximately 150 galls or internode fragments from uninfected roots were independently ground into powder in liquid nitrogen, with a mortar. Total RNA was extracted from these samples with the miRNeasy Mini Kit (Qiagen), according to the manufacturer’s instructions, with three additional washes in RPE buffer. The quality and integrity of the RNA were assessed with a Bioanalyzer (Agilent). Small RNA libraries were generated by ligation, reverse transcription and amplification (11 cycles) from total RNA (2 μg), with the reagents of the NEBNext Small RNA Library Prep Set for SOLiD. Libraries were then quantified with the Bioanalyzer High Sensitivity DNA Kit (Agilent) and sequenced on a SOLiD 5500 wildfire sequencer (Life Technologies) at the Nice-Sophia Antipolis functional genomics platform (France Génomique, IPMC, Sophia Antipolis, France).

### Bioinformatic siRNA analysis

SOLiD colour spaced reads were translated into sequences for implementation in ShortStack application. For each library, adapters were trimmed and reads matching ribosomal RNA, mitochondrial RNA were removed by performing Blast analyses with the sequences listed in the Rfam database [54]. Reads mapping on sequences corresponding to snRNA, snoRNA, mitRNA, tRNA, miRNA and pre-miRNA were removed in order to keep only siRNAs. Genomic loci accumulating siRNAs were predicted by using the ShortStack version 3.3 algorithm [41, 55]. ShortStack was run on each library separately with default parameters except: “nohp” mode (no miRNA research) and zero mismatches allowed. ShortStack software version 3.3 mapped trimmed and cleaned reads on a virtual concatenated genome composed by *A. thaliana* genome completed with plastidial and mitochondrial genomes (TAIR10.21) *and M. incognita* genome [56]. From alignments, ShortStack identified a list of coordinates of loci accumulating siRNA. Bedtools [57] with Multi-intersectbed -i option was used to find shared clusters in the different libraries. The output was filtered so that only regions that were present in at least 2 (out of 3) libraries for at least one condition either gall or uninfected root were used to serve as the final reference small RNA locus boundaries. Close clusters with a maximum distance of two nucleotides were merged with bedtools option Merge. ShortStack-count mode under default settings was then used to find relative small RNA abundance on this reference list of each library. Reads mapped on multiple loci were counted on each locus. Counts for siRNA accumulating loci from each replicate were used for differential expression analysis with the R package DESeq. Loci accumulating differentially siRNAs between galls and uninfected roots at a 5% false discovery rate (adjusted *P* value < 0.05; Benjamini Hochberg adjustment) were retrieved. Localisation of DE clusters was established in gene or in putative promoter gene region defined as the 2 kb upstream coding DNA sequence.

Sequences derived from transposable elements were searched within promoter gene region by using CENSOR algorithm [45] that screens query sequences against a reference collection of repeats. The 2 kb upstream 5’UTR of genes were retrieved and analysed by CENSOR with default parameters.

## Additional files

Additional file 1: **Figure S1.** Percentage of reads of 20, 21, 22, 23, 24 nt within each category of DicerCall clusters in gall (G7, G14) and root (R7, R14) libraries at 7 and 14 dpi. (PPTX 122 kb)

Additional file 2: **Table S1.** Characteristics of mapped reads and clusters predicted by Shortstack 3.3 in each of the three gall libraries (G1-G3) and root libraries (R1-R3) at 7 and 14 dpi. (XLSX 12 kb)

Additional file 3: **Table S2.** Selection of clusters differentially expressed (DE) in galls at 7 and 14dpi. (XLSX 12 kb)

Additional file 4: **Table S3.** Lists of DC-clusters differentially expressed in galls at 7 and 14 dpi and located within a gene body or in a promoter region. (XLSX 3559 kb)

Additional file 5: **Table S4.** Lists of DC-clusters exclusively expressed in galls or roots at 7 and 14 dpi and located within a gene body or in a promoter region. (XLSX 720 kb)

Additional file 6: **Table S5.** Number and proportion of differentially expressed (DE) DC-clusters located in a gene body or in a promoter region presented by DC categories. (XLSX 13 kb)

Additional file 7: **Table S6.** List of DC-clusters differentially expressed (DE) in galls versus uninfected roots at 7 dpi, located within promoter region of genes differentially expressed in galls at 7 dpi with inversely correlated expression profiles. (XLSX 23 kb)

Additional file 8: **Table S7** List of DC-clusters differentially expressed (DE) in galls versus uninfected roots at 14 dpi, located within promoter region of genes differentially expressed in galls at 14 dpi with inversely correlated expression profiles. (XLSX 23 kb)

Additional file 9: **Table S8.** List of DC-clusters differentially expressed (DE) in galls versus uninfected root at 7 and 14 dpi, located within promoter region of genes differentially expressed in galls at 7 and 14 dpi with inversely correlated expression profiles. (XLSX 15 kb)

## Abbreviations

AGO: Argonaute
DC: DicerCall
DCL: Dicer-like protein
DE: Differentially expressed
dpi: Days post-infection
DRM: Domains Rearranged Methyltransferase
dsRNA: Double stranded RNA
ETI: Effector-triggered immunity
GC: Giant feeding cell
hc-siRNAs: Heterochromatic siRNAs
miRNA: MicroRNA
mRNA: Messenger RNA
nat-siRNA: Natural antisense siRNA
nt: Nucleotide
phasiRNA: Phased, secondary, small interfering RNA
Pol: DNA-dependent RNA polymerase
PTGS: Post-transcriptional gene silencing
PTI: PAMP-triggered immunity
RdDM: RNA directed DNA methylation
RDRs: Dependent RNA polymerases
RISC: RNA Induced Silencing Complex
RKN: Root-knot nematodes
RPMM: Reads per million mapped reads
rRNA: Ribosomal RNA
siRNA: Small interfering RNA
sRNA: Small RNA
tasiRNA: *Trans*-acting siRNA
TE: Transposable element
TGS: Transcriptional gene silencing
tRNA: Transfer RNA
