# Supplementary material for: Characterization of siRNAs clusters in Arabidopsis thaliana galls induced by the root-knot nematode Meloidogyne incognita
Source: BMC Genomics. 2018 Dec 18;19:943. doi: 10.1186/s12864-018-5296-3 (PMC6297998; doi:10.1186/s12864-018-5296-3)
Supplement: Supplementary file 1 — Figure S1. Percentage of reads of 20, 21, 22, 23, 24 nt within each category of DicerCall clusters in gall (G7, G14) and root (R7, R14) libraries at 7 and 14 dpi. (PPTX 122 kb) [file 12864_2018_5296_MOESM1_ESM.pptx]

## Slide 1
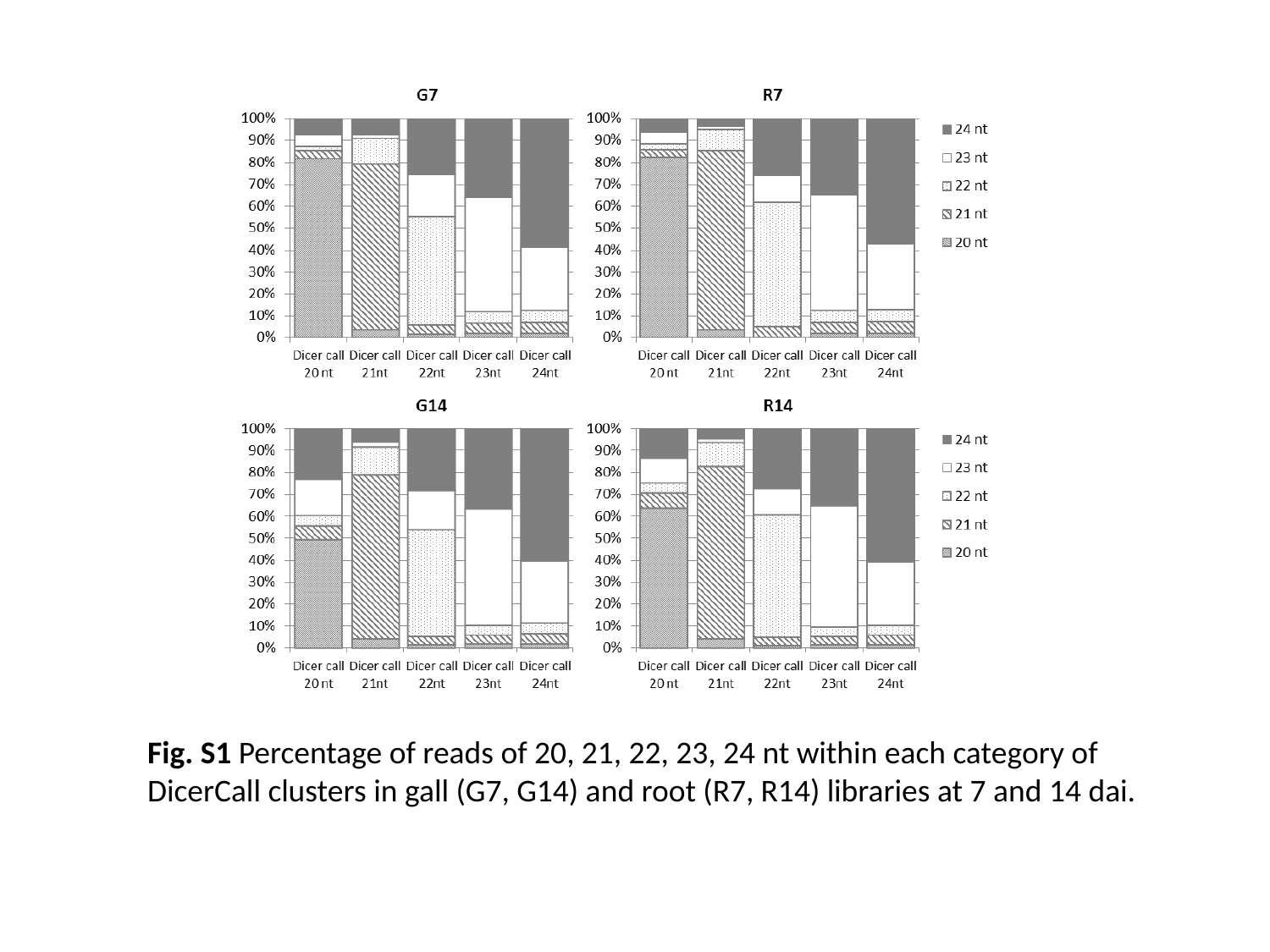

Fig. S1 Percentage of reads of 20, 21, 22, 23, 24 nt within each category of DicerCall clusters in gall (G7, G14) and root (R7, R14) libraries at 7 and 14 dai.
